# Optimisation of irinotecan dose in the treatment of patients with metastatic colorectal cancer after 5-FU failure: results from a multinational, randomised phase II study

**DOI:** 10.1038/sj.bjc.6602462

**Published:** 2005-03-08

**Authors:** E Van Cutsem, L Dirix, J-L Van Laethem, S Van Belle, M Borner, M Gonzalez Baron, A Roth, R Morant, E Joosens, G Gruia, D Sibaud, H Bleiberg

**Affiliations:** 1 University Hospital Gasthuisberg, 3000 Leuven, Belgium; 2 St Augustinus Ziekenhuis, 2610 Wilrijk, Belgium; 3 ULB Hospital, Erasme, 1070 Brussels, Belgium; 4 University Hospital Gent, 9000 Gent, Belgium; 5 Institut Med Onkologie, Inselspital, 3010 Bern, Switzerland; 6 Hopital La Paz de Madrid, 28000 Madrid, Spain; 7 Hopital de Genève, Geneva 1211, Switzerland; 8 Kantonspital St Gallen, 9007 St Gallen, Switzerland; 9 AZ Middelheim, 2020 Antwerp, Belgium; 10 Pfizer, New York, NY 10017, USA; 11 Aventis Pharma, Antony Cedex 92165, France; 12 Institute J Bordet, 1000 Brussels, Belgium

**Keywords:** colorectal cancer, CPT-11, dose optimisation, irinotecan

## Abstract

Although irinotecan 350 mg m^−2^ is a standard option for relapsed/refractory advanced colorectal cancer, there is some evidence that suggests that a higher dose may be more effective, with acceptable tolerability, following 5-fluorouracil (5-FU). This study assessed the optimal dosing strategy for irinotecan, along with treatment efficacy and safety. A total of 164 patients with metastatic colorectal cancer progressing after failure on 5-FU or raltitrexed received either 350 mg m^−2^ irinotecan (Group A; *n*=36) or 250, 350 or 500 mg m^−2^, according to individual patient tolerance (Group B; *n*=62) or based on risk factor optimisation (Group C; *n*=66). There were no complete responses. There was a trend towards a higher overall response rate in Group B (13%) than in Groups A (8%) and C (9%). Tumour control growth rate was high in all three groups: 58% in group A, 60% in Group B and 50% in Group C. A total of 34% of patients in Group B and 9% in Group C were able to receive a dose of 500 mg m^−2^. Median duration of response and time to progression were significantly longer in Groups A and B compared with Group C. No significant between-group differences for any adverse events were seen, although there was a small trend towards better tolerability in Group B. Individual dose escalation based on patient tolerance may allow more patients to receive a higher irinotecan dose without causing additional toxicity and can be an appropriate patient management strategy.

Irinotecan (CPT-11, Campto®) – a semisynthetic, water-soluble derivative of the plant alkaloid camptothecin – is the standard of care in the treatment of advanced colorectal cancer when 5-fluorouracil (5-FU)-based therapy has failed (Cunningham *et al*, 2001). Phase II trials have demonstrated objective response rates of 16–27% in pretreated patients, with stabilisation of disease in a further 40–60% of patients (Rougier *et al*, 1997; Van Cutsem *et al*, 1999). Median overall survival rates of up to 10 months are achievable when irinotecan is used in relapsed/refractory colorectal cancer (Shimada *et al*, 1993; Rothenberg *et al*, 1996, 1999; Pitot *et al*, 1997; Rougier *et al*, 1997; Van Cutsem *et al*, 1999). Two European phase III trials investigating the efficacy and safety of irinotecan, following 5-FU failure in advanced colorectal cancer, have demonstrated significant improvements in survival compared with best supportive care and 5-FU (Cunningham *et al*, 1998; Rougier *et al*, 1998). The main adverse events accompanying treatment with irinotecan in these trials were diarrhoea, neutropenia, fatigue, nausea and vomiting.

Although 350 mg m^−2^ as an intravenous infusion every 3 weeks is the standard recommended dosage of irinotecan, pharmacokinetic parameters of irinotecan-lactone and the active metabolite SN-38-lactone vary between individuals (Xie *et al*, 2002). This may be attributed to differences in the levels of the enzymes that metabolise irinotecan, notably carboxylesterase for SN-38. Furthermore, the variable interindividual patient exposure to SN-38 has been identified as an important determinant of toxicity (Mathijssen *et al*, 2002).

At the same time, there is convincing evidence of a dose–response relationship, and therefore a rationale for increasing doses when possible. In a phase I trial by Abigerges *et al* (1995), there were two recommended doses: 350 mg m^−2^ without high-dose loperamide and 600 mg m^−2^ with high-dose loperamide. With the exception of one responder treated at 260 mg m^−2^, all objective responses were observed at dose levels above 350 mg m^−2^. Merrouche *et al* (1997) provided further support for this from a phase I trial in which an increased tumour response was seen at an irinotecan dose level of 500 mg m^−2^.

Thus, these data suggest that a fixed-dose strategy for administration of irinotecan may not be optimal for all patients, thereby comprising treatment. The interindividual variability in pharmacokinetic parameters and dose–response relationship provided the rationale for investigating a dose optimisation strategy for irinotecan (Chabot *et al*, 1995). The present study investigated different strategies, using doses of irinotecan up to 500 mg m^−2^, as single-agent therapy in the treatment of patients with metastatic colorectal cancer resistant to 5-FU.

## METHODS

### Patients

Eligibility criteria included metastatic, histologically proven adenocarcinoma of the colon or rectum progressing on 5-FU-based chemotherapy (adjuvant and/or palliative); administration of ⩽2 5-FU-based regimens in the adjuvant setting or ⩽1 in the palliative setting; World Health Organization (WHO) performance status (PS) of ⩽2; adequate haematological, renal and hepatic function. Exclusion criteria included prior treatment with topoisomerase-I inhibitors; evidence of central nervous system metastases; prior history of chronic diarrhoea; current infection; or any other serious illness or medical condition.

### Study design and conduct

This was a prospective, randomised, multicentre, open-label, phase II study. The study was conducted in accordance with the Declaration of Helsinki (Hong Kong revision, 1989) and with the approval of the Ethics Committee (Institutional Review Board) at each participating centre. Written informed consent was obtained from each patient prior to his or her enrolment into the trial. An independent Monitoring Committee regularly assessed the safety and efficacy issues and reviewed the conduct of the study if needed. An External Response Review Committee (ERRC) assessed tumour responses without knowledge of the randomisation arm. The aim of the study was to determine the optimal dosing strategy in terms of efficacy and safety of single-agent irinotecan (by individual dose optimisation based on patient tolerance to treatment, or optimisation based on specific baseline risk factors) in the treatment of 5-FU-resistant patients with metastatic colorectal cancer. The primary efficacy endpoint was the overall response rate.

#### Dosing scenarios

Patients were randomised to one of three groups (A, B and C (outlined below)), each group receiving irinotecan as a 30 min intravenous infusion scheduled every 21 days. This dosing interval could be extended to a maximum of 35 days in the event of persistent toxicity to allow satisfactory recovery from the previous cycle. Doses <250 mg m^−2^ or >500 mg m^−2^ were not used in this study; patients who exhibited significant toxicity at 250 mg m^−2^ were withdrawn from the study.

Group A was the reference group in which a fixed dose of 350 mg m^−2^ of irinotecan was administered on Day 1. In subsequent cycles, the dose of irinotecan could be decreased (but not increased) according to the presence of significant toxicity at this dose.

Groups B and C investigated dosing scenarios to select patients for whom the higher dose of irinotecan (500 mg m^−2^) could be optimally used. Patients randomised to Group B received irinotecan at a starting dose of 250 mg m^−2^ followed by increasing doses (350 and 500 mg m^−2^) depending on the tolerance observed in the preceding cycle. In the event of significant toxicity, dose reductions were implemented.

In Group C, the irinotecan dose was based on protocol-defined toxicity risk factors identified at baseline: grade 3–4 neutropenia (bilirubin >70% upper limit of normal (UNL), haemoglobin <12 g dl^−1^, >3 organs involved) and/or grade 3–4 diarrhoea (PS⩾1, creatinine >70% UNL (Freyer *et al*, 2000)). Patients could be started at an irinotecan dose of 500 mg m^−2^ in the absence of toxicity risk factors. The starting dose of irinotecan was 350 mg m^−2^ in patients with one risk factor or one factor from each group, and 250 mg m^−2^ for patients with >2 risk factors or two factors from the same group. The dose was not escalated, but could be reduced to 250 mg m^−2^ in the event of significant treatment-emergent toxicity.

### Concomitant treatments and follow-up

Antiemetic drugs were administered as premedication to irinotecan infusions. Atropine was permitted for acute anticholinergic symptoms and loperamide (or similar) for delayed diarrhoea. In addition, preventative oral antibiotic therapy (e.g. an oral fluoroquinolone) was administered to patients with persistent (>48 h) grade 4 diarrhoea or for diarrhoea associated with grade 3–4 neutropenia or fever. No granulocyte-colony-stimulating factor (G-CSF) support was allowed. All patients were followed until disease progression, unacceptable toxicity or death occurred, or the patient chose to withdraw from the trial. In all cases, in each group where toxicity necessitated a dose reduction, delay or study treatment termination, the patient was followed up until the event had resolved.

### Efficacy, safety and pharmacokinetic evaluations

Tumour response rate, the primary efficacy end point, was measured according to WHO criteria and evaluated by the ERRC. Response was defined as complete (CR) plus partial (PR) response and as tumour growth control in terms of stabilisation of disease (PR plus no change/stable disease). Secondary efficacy variables were the duration of response and disease stabilisation, time to progression (TTP), time to treatment failure (TTF) and overall survival. The duration of response was measured from the first day of infusion of irinotecan to the first date that disease progression was noted or to the date of death for any reason. Time to progression was calculated from the date of randomisation to the first documented date of progression or the date of death for any reason. Time to treatment failure was the period between the date of randomisation and the date of tumour progression or treatment discontinuation for any reason. Survival was defined as the time between randomisation and death. Efficacy evaluations were performed using intent-to-treat (ITT) and per-protocol (eligible and evaluable) patient populations.

The safety population comprised all patients who had started at least one infusion of study treatment. Safety was assessed according to the National Cancer Institute Common Toxicity Criteria or, if this was not applicable, graded as mild, moderate, severe or life threatening. The safety analysis was based on the worst grade by patient and by cycle. Deaths during the trial and up to 30 days from the last infusion were recorded.

Pharmacokinetic evaluations were performed using a population approach (Chabot *et al*, 1995; Canal *et al*, 1996). At 30 min prior to infusion, and at 5 min and 3–4 h postinfusion (an additional sample was collected at 24 h postinfusion in some cases), three 5 ml blood samples (plus one predrug sample) were taken for analysis at the first cycle of chemotherapy for Groups A and C, and at the first, second and third cycles for Group B. Plasma levels of irinotecan and SN-38 were measured using reverse-phase high-performance liquid chromatography with camptothecin as an internal standard. Peak plasma concentration (*C*_max_) and the area under the plasma concentration–time curve (AUC) were calculated for both irinotecan and SN-38. In addition, total body clearance was calculated for irinotecan, and the time to reach *C*_max_ (*t*_max_) as well as the AUC normalised to l mg of irinotecan (AUC_N_) were calculated for SN-38. A three- and two-compartment model was used for irinotecan and SN-38, respectively.

### Statistical considerations

Using the hypothesis that the response rate in Groups B and C would be 20%, a total of 64 patients in each of these groups were required to yield a confidence interval (CI) band of ⩽20%. For the reference group (Group A), the number of subjects randomised was half that of Groups B and C. The 95% CIs were estimated for response, using the exact method. Confidence intervals on median values were estimated using the method described by Brookmeyer and Crowley (Simon *et al*, 1985). Descriptive statistics only were used for the pharmacokinetic parameters in each group.

## RESULTS

### Patients

A total of 164 patients entered the study: 36 in Group A, 62 in Group B and 66 in Group C (Table 1). The majority of patients (⩾97%) had received surgery and 20–30% had received radiotherapy and/or prior adjuvant chemotherapy. Based on the assessment of baseline risk factors previously described, 23 (35%) patients in Group C were assigned to receive a starting dose of 250 mg m^−2^ irinotecan, 37 (56%) patients to 350 mg m^−2^ and six (9%) patients to 500 mg m^−2^.

A total of 144 (88%) patients (31, 51 and 62 in Groups A, B and C, respectively) were eligible and evaluable for the efficacy analyses. Nine patients were ineligible due to major protocol violations (>1 line of palliative chemotherapy, and past or concurrent history of neoplasm other than colorectal adenocarcinoma in one patient) and 12 patients (not mutually exclusive) were nonevaluable for response. Early discontinuation because of adverse events rendered eight patients nonevaluable.

### Extent of exposure to irinotecan

The median dose intensity of irinotecan was similar in the three arms: 114.21 mg m^−2^ week^−1^ (95% CI 76.14–119.21) in Group A, 101.36 mg m^−2^ week^−1^ (95% CI 68.22–158.17) in Group B and 106.69 mg m^−2^ week^−1^ (95% CI 67.11–170.93) in Group C. However, the median cumulative dose was higher in Group A (1948.80 mg m^−2^) than in Groups B (1564.26 mg m^−2^) and C (1326.77 mg m^−2^), possibly due to the longer median treatment time in this group (18 weeks, compared with 16 and 13 weeks in Groups B and C, respectively).

The percentage of cycles delivered at doses of 250, 350 and 500 mg m^−2^ were as follows: 3, 92 and 0% (as this was not an option) in Group A; 41, 30 and 27% in Group B; and 33, 51 and 8% in Group C. A few cycles in each group were given at intermediate doses or at doses above 525 mg m^−2^.

In Group B, the only dose escalation option, 63% of patients had at least one dose escalation from the 250 mg m^−2^ start dose.

More than 80% of patients in each group did not require dose reduction. A total of 36–40% of patients experienced a cycle delay (Table 2). Although the majority of dose reductions in each group were made for treatment-related reasons (mostly nonhaematological adverse events across all arms), the majority of cycle delays occurred for reasons unrelated to treatment.

### Efficacy

#### Response rate

In the total (ITT) patient population (*n*=164), the overall response rates were 8, 13 and 9% in Groups A, B and C, respectively (Table 3). There were no CRs. Tumour growth control rates were higher in Groups A and B and the rates of progressive disease were lower, compared with Group C (Table 3). The pattern of response across the groups was maintained in the per-protocol (eligible and evaluable) patient population (*n*=144), with overall response rates (no CR) of 10, 16 and 10% in Groups A, B and C, respectively. Corresponding tumour growth control rates were 61, 65 and 53%.

Responses occurred at all dose levels (Table 3). However, there were only two responses at the 250 mg m^−2^ dose of irinotecan, both in Group C. Although it is difficult to interpret the data based on the small patient numbers in this study, they suggest that starting patients on a dose of 250 mg m^−2^ was not beneficial.

The median duration of response and TTP were significantly longer in Groups A and B compared with Group C (*P*=0.030) (Table 3). Despite a trend towards a shorter TTF and median overall survival in Group C, there were no significant differences across the arms for these parameters.

### Safety and tolerability

All patients were evaluable for safety. At least one adverse event was reported in all patients. However, grade 3–4 adverse events possibly or probably related to the study treatment were reported in less than half of the patients in each group (Table 4). Most of these were related to haematological or gastrointestinal (GI) events (Table 4). Grade 3–4 neutropenia with fever or infection was infrequent. Although anaemia was common, it was infrequently reported at grade 3–4 level of severity (Table 4). Diarrhoea was the most common GI event, occurring in 85% of patients, although grade 3–4 diarrhoea was less frequent (31, 21 and 27% in Groups A, B and C, respectively) (Table 4). There were no significant between-group differences for any of the adverse events reported. In addition, analysis of adverse events at the different dose levels showed no consistent evidence that toxicity increased with increasing dosage. There was no difference between the three treatment groups for the number of patients reporting ⩾1 grade 3–4 adverse event considered to be possibly or probably treatment-related (Group A, 42%; Group B, 48%; Group C, 49%). Overall, 74 serious adverse events considered possibly or probably related to study medication occurred in 39 patients.

### Treatment discontinuations

At the designated study end date, 159 (96.95%). patients had discontinued treatment (Group A, 97%; Group B, 95%; Group C, 99%) (Table 5). Disease progression resulted in proportionately fewer discontinuations in Group B (57%) than in Groups A (72%) and C (80%), and included fatalities arising from progressive disease (one patient in each of Groups A and B, and two patients in Group C). There was also one fatality: a case of aspiration pneumonia secondary to vomiting in a patient in Group B receiving the first cycle of irinotecan 250 mg m^−2^. Five (42%) of the patients who discontinued treatment from Group B were receiving the 250 mg m^−2^ dose option during cycle 1 at the time of withdrawal. Adverse events leading to discontinuations are listed in Table 5.

### Pharmacokinetic parameters

The principal pharmacokinetic parameters for irinotecan and SN-38 measured in 29 assessable patients are presented in Table 6. The mean total body clearance values of irinotecan were similar across all three groups and no relevant differences in dose-normalised exposure were seen. Exposure to irinotecan and SN-38 increased proportionally over the 250–500 mg m^−2^ irinotecan dose range. In the population pharmacokinetic analysis, exposure to irinotecan appeared to be increased in patients with PS 1 or 2, and in patients with high alkaline phosphate levels.

## DISCUSSION

The results of this phase II study confirm the activity of single-agent irinotecan in patients with metastatic colorectal cancer who have failed previous therapy with 5-FU. All three treatment strategies investigated were active and demonstrated acceptable tolerability patterns. Although almost all patients in the study had ⩾1 adverse event, less than half of the patients in each treatment strategy had grade 3–4 toxicity.

The main aim of this study was to determine the optimal irinotecan dosing regimen for the treatment of this population, with the primary end point being response rate. The highest overall response rate was seen in patients in Group B (13%). In this group, four (21%) of the 19 patients receiving irinotecan 500 mg m^−2^ achieved a response. There was little difference in the overall response rates in Groups A and C (8 and 9%, respectively). An interesting observation in this study was the relatively higher rate of progressive disease in Group C (44%) compared with Groups A and B (36 and 31%). None of the differences in response rate between the groups were statistically significant. It is worth mentioning that the response rate observed in Group A was unusually low, and less than that seen in published studies of similar populations of patients treated with the same schedule (Rougier *et al*, 1997; Van Cutsem *et al*, 1999). This may be due to changes in first-line treatment that have occurred in recent years; compared with patients treated in earlier studies, those in the present study may have been more heavily pretreated with 5-FU and oxaliplatin in the first-line setting, thus making them more chemotherapy resistant. Despite the lower response rate in Group A, it is within the CIs of previous studies and so can be considered representative.

The lack of a significant difference in overall response rates between the groups may reflect the fact that the median dose intensity of irinotecan delivered was relatively constant across the three groups, despite a proportion of patients in Groups B (34%) and C (9%) receiving an irinotecan dose of 500 mg m^−2^. This finding is probably due mainly to the fact that a disproportionate number of patients (more than one-third) in each of Groups B and C never received a dose of more than 250 mg m^−2^, and so were possibly underdosed. The likelihood of underdosing in Groups B and C is supported by the observation that only 6% of patients in Group A required dose reduction from 350 to 250 mg m^−2^.

There were no significant differences between Groups A and B in TTP or overall survival. This may be due to an insufficient powering of the study and/or too small a difference in response rates. A previous meta-analysis conducted in patients with advanced colorectal cancer reported that only large differences in response rate correspond to a significant difference in TTP (Buyse *et al*, 2000). Both TTP and duration of response were significantly shorter in Group C than in Groups A and B, and there was also a trend for a shorter overall survival in this group. The relatively poor efficacy seen in Group C may have been due to a combination of underdosing (i.e. a significant number of patients receiving irinotecan 250 mg m^−2^) and the small number of patients who received the high dose of irinotecan (500 mg m^−2^) (six patients or 9%).

There was a trend towards a better safety profile in Group B. Grade 3–4 neutropenia was 31% in Group B, 47% in A and 44% in Group B. Similarly, there was less grade 3–4 diarrhoea in Group B as compared with Groups A and C (21 *vs* 31 and 27%, respectively), despite 34% of patients receiving the highest irinotecan dose. We cannot exclude the contribution to this difference of imbalances in gender ratio (more male patients in Group B) and PS (more patients with PS=0 in Group B). However, it is possible that the results reflect the aim of the strategy adopted in Group B, which was to avoid subjecting patients to higher doses than they were able to tolerate. Indeed 10 out of 12 patients in Group B who withdrew from the study due to treatment-related adverse events received the lowest dose of irinotecan (250 mg m^−2^) and therefore would not have tolerated an increased dose of irinotecan. However, it should also be noted that severe toxicity leading to treatment discontinuation occurred more frequently in Group B despite the low dose given to all patients in the first cycle. In Group C, despite the strategy of basing the initial irinotecan dose on predetermined risk factors, patients administered the 250 mg m^−2^ dose demonstrated higher rates of grade 3–4 anaemia and diarrhoea compared with those receiving the 350 and 500 mg m^−2^ doses.

This study demonstrates that intrapatient dose escalation based on toxicity in the preceding cycle dose, as practised in Group B, is feasible. Although the increase in the response rate over the whole group was modest compared with the standard irinotecan dose, clinical benefit may be seen in those patients who are able to receive 350 and 500 mg m^−2^, which, in this study, was associated with a response rate of 25 and 21%, respectively. The findings of our study in pretreated patients are in agreement with those of a nonrandomised study in previously untreated patients (Ychou *et al*, 2002): the greater proportion of patients who are able to receive the higher dose and the higher response rate achieved in the latter study compared with our study is probably a reflection of interstudy differences in the starting dose, dose escalation guidelines and in the study population (previous treatment compared with no previous treatment).

In contrast with the feasibility of the strategy in Group B, the use of dose optimisation according to the baseline risk characteristics identified in our study protocol (as practised in Group C) appeared not to be an appropriate approach. This may be because the risk characteristics identified were not valid in this setting or that the algorithm for dose assignment was not relevant. Further investigation is required to clarify this.

In conclusion, the data from our randomised phase II study suggest that individual dose optimisation based on toxicity in the preceding cycle is feasible and merits further investigation. Increasing the dose of irinotecan to 500 mg m^−2^ can be of benefit in selected patients. It will be necessary to identify the most appropriate starting dose, as the dose of 250 mg m^−2^ used in this study was possibly too conservative. Data from pharmacogenomic research are likely to be useful in the future for identifying the most appropriate starting dose of irinotecan for individual patients.

## Figures and Tables

**Table 1:** Patient demographics and baseline characteristics

	**Treatment group**		
	**A**	**B**	**C**
Number of patients (*n*); randomised (eligible and evaluable)	36 (31)	62 (51)	66 (62)
Gender; male : female (%)	50 : 50	71 : 29	62 : 38
Age in years; median (range)	60 (29–71)	59 (33–70)	60 (30–70)
Weight loss at baseline in relation to usual body weight (% of population)			
⩽5%	88.9	85.5	87.9
>5%	5.6	4.8	3.0
Unknown	5.6	9.7	9.1
Mean loss (kg)	1.1	0.9	1.0
WHO PS			
Median	1	0	1
0 (%)	50.0	59.7	45.5
1 (%)	44.4	35.5	53.0
2 (%)	5.6	4.8	1.5
Primary tumour location			
Colon	63.9	66.1	66.7
Rectum	36.1	33.9	33.3
Number of organs with metastatic involvement; median (range)	2 (1–3)	2 (1–3)	2 (1–4)
Synchronous metastases (%)	41.7	59.7	56.1
Sites of metastatic disease (%)			
Liver	69.4	79.0	80.3
Liver alone	48.0	53.1	37.7
Liver and other organs	52.0	46.9	62.3
Lung	41.7	30.6	31.8
Peritoneum	11.1	4.8	13.6
Lymph nodes	11.1	21.0	22.7
Colon	0	6.5	1.5
All others^a^	27.8	22.6	30.3
Median (range) time to randomisation (months) from			
First diagnosis	18.1 (4.7–82.3) (*n*=35)	12.7 (3.0–76.3) (*n*=61)	12.6 (3.2–160.1) (*n*=66)
First metastasis	9.1 (0.0–54.7) (*n*=35)	9.0 (0.6–42.7) (*n*=62)	8.1 (0.1–51.6) (*n*=65)
Prior anticancer treatment (% of patients)			
Surgery	97.2	98.4	97.0
Radiotherapy	30.6	21.0	22.7
Adjuvant chemotherapy	33.3	25.8	21.2
At least one symptom at baseline			
(% of patients)	72.2	62.9	77.3
At least one abnormal laboratory value at baseline (% of patients)	97.2	95.2	93.9

a Soft tissue, bone, adrenal, pelvis, abdomen, pleura, retroperitoneum, spleen, mediastinum, skin.

WHO, World Health Organization.

**Table 2:** Extent of exposure to irinotecan

	**Treatment group**		
	**A**	**B**	**C**
Number of patients exposed	36	62	66
Number of treatment cycles	216	370	333
Median (range) number of cycles	6 (1–24)	5 (1–21)	4 (1–15)
Median (range) treatment duration (weeks)	18 (3–78)	16 (3–64)	13 (3–46)
Cycles by dose (% of cycles)^a^			
250 mg m^−2^	3	41	33
350 mg m^−2^	92	30	51
500 mg m^−2^	—	27	8
Median actual dose intensity (mg m^−2^ week^−1^) (95% CI)	114.21 (76.14–119.21)	101.36 68.22–158.17)	106.69 (67.11–170.93)
Median cumulative dose (mg m^−2^) (95% CI)	1948.80 (314.65–8373.08)	1564.26 (247.52–10 100.00)	1326.77 (249.73–4899.13)
At least one dose increase (% of patients)	—	63	—
At least one dose reduction^b^			
% of patients	17	15	17
% of cycles	4	3	5
At least one cycle delayed^b^			
% of patients	36	40	36
% of cycles	19	15	15

a Some cycles were administered at intermediate doses.

b For any reason (see text).

**Table 3:** Efficacy results

**Parameter**	**Group A (*n*=36)**	**Group B (*n*=62)**	**Group C (*n*=66)**	***P*-value^a^**
Overall response rate, % (95% CI)^b^	8 (1.8–22.5)	13 (5.7–23.9)	9 (3.4–18.7)	
Overall response rate, % (95% CI)^b^ Per protocol	10 (2.0–25.8)	16 (7.0–28.6)	10 (3.6–19.9)	
250 mg m^−2^ c	—	(0/16) 0%	(2/20) 10%	NC
350 mg m^−2^ c	(3/31) 10%	(4/16) 25%	(4/36) 11%	NC
500 mg m^−2^ c	—	(4/19) 21%	(0/6) 0%	NC
Tumour growth control rate (%)	58%	60%	50%	NC
Progressive disease (%)	36%	31%	44%	NC
Median duration of response (months)	6.4	6.6	4.3	0.03
Median TTP (months)	4.1	4.2	3.0	0.019
Median TTF (months)	3.7	3.4	2.5	NS
Median overall survival (months)	12.5	12.1	10.9	NS

Results are presented for the ITT population, unless otherwise stated.

a A *vs* C and B *vs* C.

b There were no CRs.

c Response rate is expressed as a percentage of patients treated at that dose level as their highest dose in each group.

CI, confidence interval; NC, not calculated; NS, not significant.

**Table 4:** Adverse events

**Grade 3–4 adverse events^a^**	**Treatment group: *n* (% of patients)**		
	**A (*n*=36)**	**B (*n*=62)**	**C (*n*=66)**
At least one grade 3–4 adverse event^a^	15 (42)	30 (48)	32 (48)
Haematological			
Leukopenia	9 (25)	15 (24)	21 (32)
Neutropenia	17 (47)	19 (31)	29 (44)
Anaemia	3 (8)	1 (2)	5 (8)
Infection (grade 3–4 neutropenia present)	2 (6)	0	2 (3)
Fever without infection (grade 3–4 neutropenia present)	0	2 (3)	3 (5)
Gastrointestinal (GI)			
Vomiting	5 (14)	10 (16)	6 (9)
Diarrhoea	11 (31)	13 (21)	18 (27)
Nausea	4 (11)	7 (11)	7 (11)
All other GI events^b^	5 (14)	5 (8)	4 (6)
Other adverse events			
Fatigue	3 (8)	7 (11)	8 (12)
Fever (grade 3–4 neutropenia absent)	0	1 (2)	3 (5)
Infection (grade 3–4 neutropenia absent)	2 (6)	1 (2)	3 (5)

a Possibly or probably related to study treatment.

b Anorexia, five (3%) cases; cholinergic syndrome, three (2%) cases; GI pain, two (1%) cases; dehydration, three (2%) cases; stomatitis, one (1%) case.

**Table 5:** Patient discontinuations

	**Treatment group: *n* (% of patients)**		
	**A (*n*=36)**	**B (*n*=62)**	**C (*n*=66)**
No. of patients still on treatment at cutoff date	1 (3)	3 (5)	1 (2)
Total treatment discontinuations	35 (97)	59 (95)	65 (99)
*Nonfatal reasons*			
Progressive disease	25 (69)	34 (55)	51 (77)
Treatment-related adverse event	2 (6)	12 (19)	6 (9)
Adverse events leading to discontinuation^a^			
Fatigue	1 (3)	3 (5)	2 (3)
Vomiting	1 (3)	3 (5)	2 (3)
Diarrhoea	—	4 (7)	2 (3)
Nausea	—	2 (3)	2 (3)
Neutropenia	—	2 (3)	1 (2)
Febrile neutropenia	—	2 (3)	—
Neutropenic infection	1 (3)	—	—
Infection	—	—	2 (3)
Fever (infection absent)	—	1 (2)	—
All other nonfatal events^b^	—	5 (8)	1 (2)
Patient refusal	1 (3)	4 (7)	1 (2)
Other	6 (17)	7 (11)	4 (6)
*Fatal reasons*			
Death due to treatment-related adverse events	—	1 (2)	—
Death due to progressive disease	1 (3)	1 (2)	2 (3)
Cardio-respiratory failure	—	—	1 (2)

a Not mutually exclusive. Patients may have discontinued treatment for more than one adverse event reason.

b Group B: aggravation reaction, two (3%) cases; anorexia, one (2%) case; dehydration, one (2%) case; small bowel obstruction, one (2%) case. Group C: anorexia, one (2%) case.

**Table 6:** Pharmacokinetic profiles of irinotecan and SN-38 at different doses of irinotecan

	**Arm A**	**Arm B**			**Arm C**	
Cycle	1	1	2	3	1	1
No. of patients	6	13	8	5	5	5
Dose (*n*) (mg m^−2^)	350	250	350	300 (1)/500 (4)	250	350
*Irinotecan*						
Infusion duration (h)	0.5–1.5	0.5–1.5	0.5–1.0	0.5–1.1	0.5–1.6	0.5–1.1
*C*_max_ (mg l^−1^)	5.88 (4.79–9.18)	4.55 (3.05–5.87)	6.12 (5.33–6.70)	8.40 (4.57–8.62)	3.61 (3.26–4.56)	7.13 (5.10–7.79)
AUC (mg h l^−1^)	32.7 (14.3–36.4)	20.5 (11.6–30.9)	27.8 (20.4–39.2)	44.7 (28.0–50.6)	19.7 (15.3–29.0)	33.4 (22.9–46.3)
Clearance (l h m^−2^)	9.3 (8.4–21.3)	10.6 (7.0–18.9)	10.9 (8.2–14.9)	9.1 (5.9–15.6)	10.6 (7.53–14.4)	9.03 (6.55–13.3)
*SN-38*						
Median *t*_max_ (h)	0.7 (0.6–1.5)	0.6 (0.5–1.6)	0.6 (0.5–1.0)	0.7 (0.6–1.1)	1.5 (0.6–1.6)	1.0 (0.6–1.1)
*C*_max_ (μg l^−1^)	61.9 (33.5–86.7)	49.7 (24.0–138.0)	58.7 (38.0–168.0)	80.7 (34.9–97.3)	40.6 (33.9–962)	67.9 (50.2–135)
AUC (μg h l^−1^)	668 (362–1110)	676 (324–1140)	960 (546–1300)	1420 (609–1610)	595 (403–903)	768 (579–1395)
AUC_N_^a^ (μg h l^−1^)	1.9 (1.1–2.9)	2.4 (1.3–5.0)	2.6 (1.6–4.4)	2.6 (1.3–5.1)	2.0 (1.5–3.2)	2.5 (1.5–4.4)

a Normalised to 1 mg irinotecan dose.

Data are expressed as median (95% CI) unless otherwise stated.

AUC, area under the plasma concentration–time curve; *C*_max_, maximum plasma concentration; *t*_max_, time to reach maximum plasma concentration.
